# ﻿Daily and seasonal time partitioning in surface activity of *Porcellioalbinus* (Isopoda, Oniscidea) in the arid region of Zarat (Gabes, Tunisia)

**DOI:** 10.3897/zookeys.1101.73834

**Published:** 2022-05-18

**Authors:** Lamia Medini-Bouaziz, Sonia Hamaied, Ahmed Ouni, Mohamed El Gtari

**Affiliations:** 1 Laboratoire Diversité, Gestion et Conservation des Systèmes Biologiques, LR18ES06, Faculté des Sciences de Tunis, Université de Tunis El Manar, 2092 Manar II, Tunis, Tunisia Université de Tunis El Manar El Manar Tunisia

**Keywords:** Burrowing species, circadian rhythm, desert, epigeic activity, woodlice

## Abstract

The terrestrial isopod *Porcellioalbinus* is a burrowing species, dwelling in the desert of south Tunisia. Field studies were carried out in the coastal area of Zarat, Tunisia, to examine the surface activity rhythm of *P.albinus* in relation to daily and seasonal variations in environmental conditions. The activity of *P.albinus* was followed once a month, from November 2012 through October 2013. Hourly capture frequency was compared across the different seasons of the year. *Porcellioalbinus* is a strictly nocturnal species showing a nycthemeral rhythm regulated by the rhythmic and natural variations of the duration of the dark period. A positive correlation is observed between the circadian rhythm of the locomotor activity of the species and the duration of the dark period outside its burrow, *P.albinus* has a single daily activity peak. Individuals concentrated their activity in the first part of the night in winter and in the second part in summer. This peak is more spread out in spring and autumn. The differences in the activity rhythm of *P.albinus* between different seasons may be determined by two important factors, namely temperature and the length of the dark period.

## ﻿Introduction

Activity patterns in terrestrial organisms are crucial for migration, survival, and reproduction (Engenheiro et al. 2005). Hence, it is an important and relevant ecological parameter. Terrestrial isopods are an important component of the soil macrofauna and are restricted in their activity in space and time to conditions where air and substrate moisture are relatively high. Therefore, terrestrial isopods are mainly nocturnal and show strong seasonal patterns in activity, especially in arid regions. At night they appear from their hiding places and are able to wander in dry areas where they are not usually found during the day. In general, this is true for the majority of terrestrial isopods, but there are exceptions such as *Armadillidiumvulgare* Latreille, 1804 and *Hemilepistusreaumurii* (Milne-Edwards, 1840), which are more resistant to desiccation compared to other species (Edney 1951; Ayari et al. 2016), and can appear out of their burrows in full sunlight (Cloudsley-Thompson 1951; Ayari et al. 2018). Extensive and detailed studies on these activity patterns in different species of terrestrial isopods unravel species-specific activity patterns deviating from strict nocturnal activity. For example, the semi-terrestrial burrowing isopod *Tylosspinulosus* Dana, 1853 shows activity along the beach depending on the tide (Jaramillo et al. 2003); *Armadillidiumvulgare* is active in the morning hours in open habitats (Cloudsley-Thompson 1951) and the forest species *Porcelliumconspersum* (C. Koch, 1841) is active mainly during the day in spring (Tuf and Jeřábková 2008). *Hemilepistusreaumurii* occurs in desert regions and is active mostly during the hours of daylight with a bimodal pattern during warm months and a unimodal surface activity pattern during cold months (Ayari et al. 2018). The daily epigeic activity of terrestrial isopods depend mainly on the light-dark regime and to a lesser extent on air humidity and temperature (Tuf and Jérabkova 2008).

Living often alongside *H.reaumurii* (Medini-Bouaziz et al. 2017a), the burrowing desert isopod *Porcellioalbinus* Budde-Lund, 1885 is one of the few terrestrial isopod species well adapted to the sandy desert habitats of North-Africa (Monod 1932; Medini-Bouaziz 2002). Despite its wide distribution, there have been few studies on this species’ ecology. Only Linsenmair (1989, 2007) provided some information on its reproduction and behaviour, while the density and the distribution of *P.albinus* burrows were studied by Fraj et al. (2008) in the Kebili region (Tunisia). Recently, Medini-Bouaziz (2018) and Medini-Bouaziz et al. (2017a, b) carried out field studies on the abiotic factors determining the spatial distribution of burrows, the population dynamics, and the reproductive aspects of *P.albinus* in the Zarat area in Tunisia. Previous studies on behavioural and reproductive strategies of *Porcellio* species (Medini-Bouaziz et al. 2017b; Medini-Bouaziz 2018) in the Zarat region showed that specimens of *P.albinus* were observed outside their burrows at night and never during the day in contrast to *H.reaumurii*. However, these patterns have never been studied in detail. Therefore, we aim to investigate the nocturnal rhythm of the species and the environmental factors that influence it. We investigated the time partitioning of surface activity during night hours and its variation across the seasons to understand the surface activity rhythm of *P.albinus* under natural conditions and contribute to the knowledge of this widespread species’ ecology.

## ﻿Materials and methods

### ﻿Study area

The study site is located in the sandy coastal area of Zarat, south of Gabès, Tunisia (33°40'N, 10°21'E (DDM)). The area is dominated by *nebkas*, morphological structures that were formed following an accumulation of sand brought by the wind and trapped by an obstacle (Medini-Bouaziz et al. 2017a). This area is situated at 1.5 km from the coastline. The climate is arid, i.e., rainfall is irregular and ranges between 100 and 200 mm per year (Genin et al. 2006), temperatures are usually high with considerable seasonal variation. The proximity of the study area to the sea moderates summer temperatures and therefore they do not exceed 28 °C on average in summer and 13 °C in winter (Genin et al. 2006).

### ﻿Terrestrial isopod activity and environmental variables

Seasonal activity of *P.albinus* was investigated in the field in winter 2012, spring, summer, and autumn 2013. Recordings were performed at night in a rectangular area of 1,000 m^2^ (100 m × 10 m) which was subdivided into ten corridors of 10 m × 1 m. Sampling was done one day every month for 24 hours. Sampling took place every two hours between and incorporating sunset and sunrise starting at 16 h in winter and spring and 17 h in summer and autumn. White flashlights were used to count and capture the individuals of *P.albinus*, while walking back and forth in every corridor. All individuals captured were used for a study on the reproductive cycle of *P.albinus* (see Medini-Bouaziz et al. 2017b). We are aware that this could lead to a small underestimation of the activity patterns at later time step during the night, but since activity periods of terrestrial isopods are relatively short, we do not expect that this could have a large impact on the results (Den Boer 1961). All ten corridors could be sampled in one hour. Only individuals whose size reached or exceeded 8 mm were counted and captured, those whose size is less than 8 mm (equivalent to less than 2 months old) do not leave their burrows (Medini-Bouaziz et al. 2017b).

We calculated the duration of surface activity as the period between the first and the last observed individual per night. This study was carried out simultaneously with the study from Medini-Bouaziz et al. (2017b) on population dynamics and reproduction of *P.albinus*.

At the same time, the most important environmental parameters (average temperature, wind speed, air humidity, dew point, and cloud cover) were registered every two hours, based on weather data for the Zarat region provided by the AccuWeather Az-Zarat (2013) at the day of sampling.

*Porcellioalbinus* surface activity is assessed by its capture frequency which corresponds to the ratio between the number of individuals collected per time slot and the total number of individuals collected for all time slots.

### ﻿Statistical analysis

To study the variation in surface activity between seasons, we used analysis of variance (ANOVA). We used Principal Component Analysis (PCA), with surface activity of *P.albinus* during the night as response variable to understand the importance of temporal (season and time) and environmental variables (temperature, wind speed, moisture, dew point and cloud cover) for *P.albinus* surface activity at night. All the statistical tests were applied at a confidence level (p-value) of 0.05 and performed using the XLSTAT 2018.6 software, used as a Microsoft Excel plug-in. A Pearson correlation test was used to investigate the strength of the linear relationship between length of the surface activity of *P.albinus* and the length of the dark period. A Friedman test is used to test the inter-seasonal difference observed in the duration of the surface activity period of *P.albinus*.

## ﻿Results

### ﻿Daily surface activity

**Figure 1.**
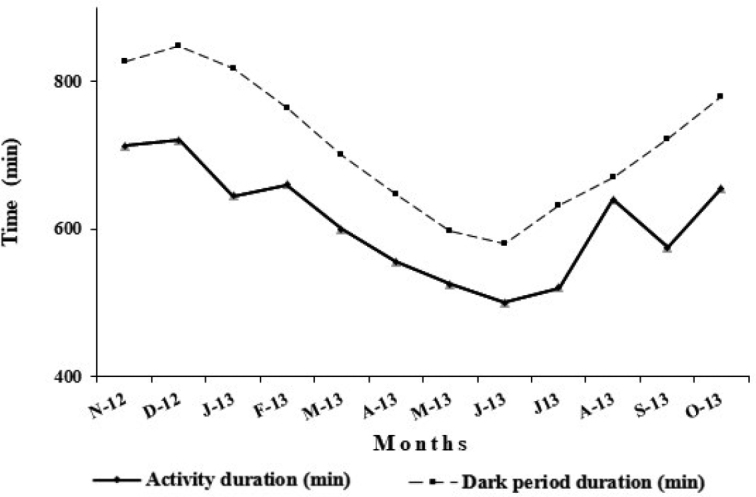
Daily surface activity variation of *Porcellioalbinus*. Surface activity deduced from the mean time interval between the first and last observed isopod.

The length of the surface activity of *P.albinus* was correlated with the length of the dark period (r = 0.874, p < 0.05) (Fig. 1). During the whole sampling period from November 2012 through October 2013, *P.albinus* showed strict nocturnal activity: individuals, whose size is equal to or exceeds 8 mm (Medini-Bouaziz et al. 2017b), began to emerge from their burrows after dusk (26 ± 4 min in March 2013 to 106 ± 6 min in September 2013) and returned to their shelters before dawn (17 ± 5 min in July 2013 to 110 ± 3 min in January 2013). The duration of the nocturnal surface activity of *P.albinus* varied from month to month. The shortest activity period and longest activity period were respectively in June and December coinciding with the shortest and longest dark period (Fig. 1).

### ﻿Seasonal surface activity

**Figure 2.**
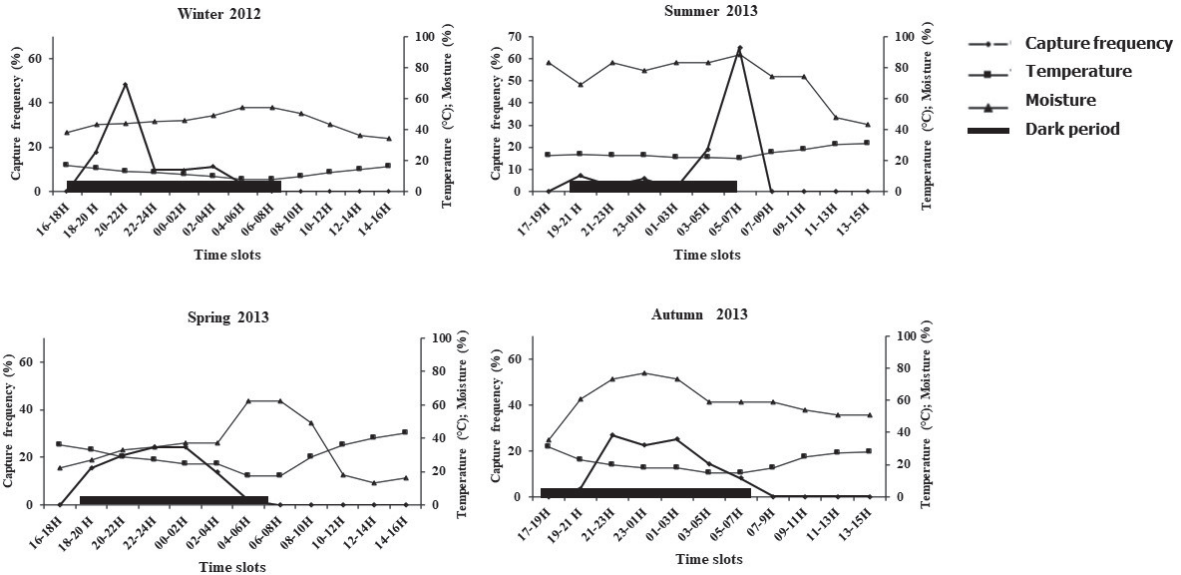
Temporal distribution of the surface activity of *P.albinus* during the four seasons in relation to the time of the day. Surface activity was deduced from the number of captured individuals. The continuous thick horizontal line indicates the dark period.

In total, 317 individuals of *P.albinus* were recorded and collected. The surface activity of this species shows a surface activity period that is longer in winter (675.0 ± 39.7 min) than in the other seasons, but this difference is statistically insignificant (Table 1). In winter, *P.albinus* concentrated its activity in the first part of the night, when the temperature was high enough, reaching its maximum at ~ 21:00 h (Fig. 2). In summer, *P.albinus* shifted its activity to the second part of the night, with a peak at ~ 05:00 h when temperature decreased. In spring and autumn, *P.albinus* surface activity was evenly distributed throughout the night. Table 2 shows the dependence of frequency on temperature, moisture, dew point, and wind speed. Capture frequency did not depend on Cloud cover or amount of rainfall.

**Table 1.** Coefficient of regression (R) below the diagonal and p values above the diagonal for pairwise comparison of *P.albinus* seasonal activity duration between the different seasons.

	Winter	Spring	Summer	Autumn
**Winter**	0	0.586	0.232	1.000
**Spring**	-1.333	0	0.921	0.586
**Summer**	-2.000	-0.667	0	0.232
**Autumn**	0.000	1.333	2.000	0

**Table 2.** Results of the Fiedman test investigating the dependency of capture frequency on different environmental variables.

	Temperature (°C)	Moisture (%)	Wind speed (Km/h)	Cloud cover (%)	Dew point (°C)	Rain (%)
R^2^	0.5202	0.6330	0.3233	0.1884	0.9305	0.2000
F	10.1211	16.0993	4.4595	2.1666	125.0361	2.3333
p value	**0.0001**	**< 0.0001**	**0.0111**	0.1143	**< 0.0001**	0.0955

**Figure 3.**
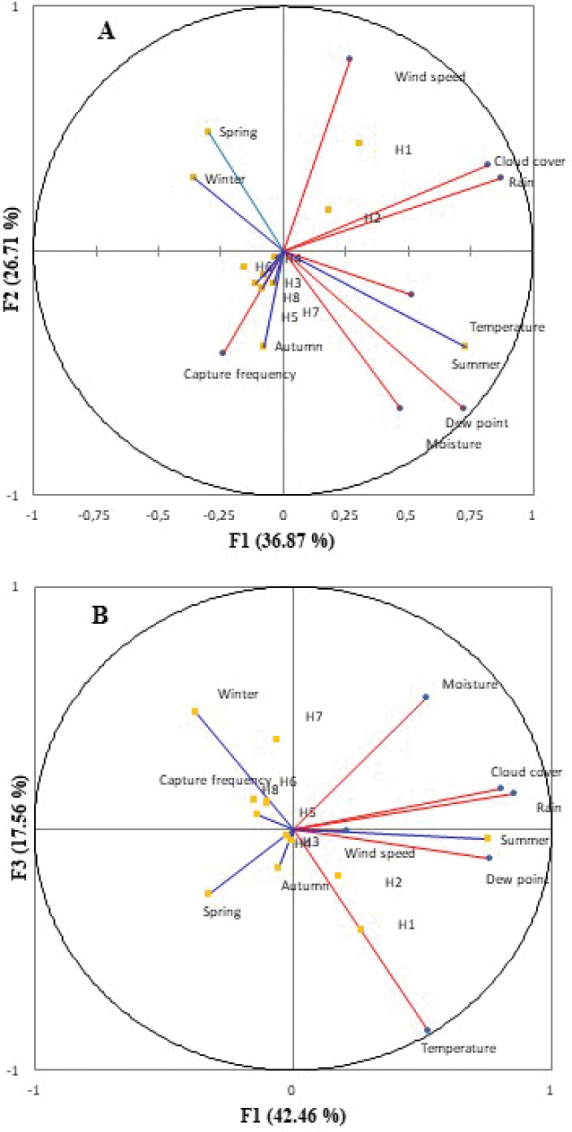
Ordination Biplot of the Principal Correspondence Analysis applied on the environmental parameters measured in each season for studied time slots of *Porcellioalbinus* locomotor activity. H1 (16 h–18 h); H2 (18 h–20 h); H3 (20 h–22 h); H4 (22 h–00 h); H5 (00 h–2 h); H6 (2 h–4 h); H7 (4 h–6 h); H8 (6 h–8 h).

The Principal Component Analysis (PCA) gives more insight into the important environmental variables that influence capture frequency and thus surface activity of *P.albinus*. The first (F1) and second (F2) PCA axes explain 42.46% and 29.51% of the variance respectively with a cumulative percentage of 71.98 (Fig. 3A). Almost half of the variables are correlated to F1. Cloud cover, dew point, rain, and temperature as well as the effect of the summer season are projected positively along F1. Along F2, wind speed, the first trapping hour (H1) and the effect of the spring season are positively correlated; on the other side, capture frequency, the effect of the autumn season, and moisture are negatively correlated along F2. The effect of the winter season and temperature strongly correlates to F3 (Fig. 3B) respectively positive and negative. The positive correlation between temperature and summer and the negative correlation between temperature and winter are probably important to explain the difference observed in capture frequency between winter and summer.

## ﻿Discussion

The desert species *P.albinus* survives extreme heat and drought in desert regions thanks to the microclimate within the burrows they dig, analoguous to the behaviour of *H.reaumurii* (Medini-Bouaziz 2018; Linsenmair 1975; Hoffmann 1983). The burrows are dug according to standards that consider the prevailing wind direction, the geo-environmental conditions, and the type of vegetation covering the nebkas (Medini-Bouaziz et al. 2017a). During the night, larger specimens (> 8 mm in body length) emerge from their burrow to forage (Medini-Bouaziz et al. 2017b). The absence of specimens less than 8 mm outside burrows could be explained by parental care during the first two months of the life of juveniles which is a sensitive period for the species’ survival (Medini-Bouaziz et al. 2017b). Specimens of *P.albinus* were active every night except during rainy nights. Despite its scarcity in desert environments, rainfall can play an important role in regulating the activity of *P.albinus*, which remains inside the burrow for days after rain falls and shows no activity outside its shelter until the sand is completely dry (Medini-Bouaziz 2018). This could be explained by the fact that *P.albinus*, when leaving its burrow for foraging, piles up sand from the burrow in front of the opening (Linsenmair 2007). This sand, marked by the owner’s individual chemical signature, is then used as a landmark to find its burrow back when returning (Linsenmair 2007; Medini-Bouaziz et al. 2017a).

In *P.albinus*, there must be an innate 24-hour rhythm controlled by the rhythmic and natural variations of the duration of the dark period since animals only emerge from their burrows during the dark period. *Porcellioalbinus* exhibits a different activity rhythm compared to *H.reaumurii*, although the two species are from similar guild of burrowing species and occupy the same habitat in Zarat. *Hemilepistusreaumurii* shows diurnal surface activity (Cloudsley-Thompson 1956a) from February to November and remains inactive during December and January (Ayari et al. 2018). Nocturnal activity as observed in *P.albinus* is conventional for the majority of terrestrial isopods, while diurnal activity of *Hemilepistus* is an evolutionary novelty. The reason for the shift to daytime activity in *H.reaumurii* could be to avoid competition with other burrowing species such as *P.albinus* (Jaramillo et al. 2003), but also to avoid predation pressure by scorpions.

A nocturnal and photonegative lifestyle could be explained by the species’ water balance. *Porcellioalbinus* is a drought-sensitive species with a high rate of water loss through transpiration (Cloudsley-Thompson 1956b). The reverse is true for *H.reaumurii*, a species with a low transpiration rate and a thick cuticle (Ayari et al. 2016). Thus, the degree of nocturnal activity of terrestrial isopods is correlated with the ability to withstand water loss by transpiration.

Interseasonal comparisons of capture frequencies show that the epigeic activity of *P.albinus* in Zarat is high at the beginning and the end of the dark period in winter and summer, respectively. This nocturnal peak is more spread out in spring and autumn and occurs in the middle of the night. In general, most of the variability recorded in daily activity patterns in terrestrial isopod activity could be explained by habitat type and season. Tuf and Jérabkova (2008) found different activity patterns across seasons and between closed forests and clear-cut areas. Terrestrial isopods are known to cope with differing environmental conditions in their habitat by adopting various patterns of activity in response to these conditions (Warburg et al. 1984). Among littoral isopods, nocturnal species such as the genus *Ligia* or *Tyloseuropeus* Arcangeli, 1938 (Colombini et al. 1996) prefer high humidity (Warburg et al. 1984; Jaramillo et al. 2003). Terrestrial isopods from mesic habitats (such as the genera *Oniscus*, *Porcellio*, and *Armadillidium*) responded less strong to light and humidity compared to littoral species.

Although the number of different forms of activity, and the quantitative relationships between these forms are correlated with air humidity (see, e.g., Den Boer (1961) for *Porcellioscaber* Latreille, 1804), in xeric habitats, all isopods are positively hydrokinetic and negatively photoreactive (except for *H.reaumurii*) with a drop in activity at high temperatures (Warburg 1968). However, temperature and humidity were not the only factors affecting surface locomotor activity of *P.albinus* and the interseasonal variability observed could also be explained by wind speed negatively affecting the capture frequency. Similar results have been recorded in *Oniscusasellus* Linnaeus, 1758 and *Porcellioscaber* where the number of wandering isopods on the surface decreased with increasing wind speed (Cloudsley-Thompson and Cupta 1960). Of course, environmental variables such as wind speed could also indirectly influence temperature and humidity.

In conclusion, *P.albinus* is a nocturnal species showing activity patterns depending upon temperature, humidity, and related environmental variables. These activity patterns also strongly depend on the season with a strong peak in activity during the early night in winter and towards the end of the night in summer when temperatures and humidity are most favourable while activity is more evenly distributed throughout the night during spring and autumn.
